# Ameliorative effects of avocado oil on bleomycin‐induced lung fibrosis and oxidative stress in rats

**DOI:** 10.14814/phy2.70228

**Published:** 2025-02-04

**Authors:** Naoures Ochi, Anouar Abidi, Wael Taamalli, Ayda Ayedi, Hichem Sebai

**Affiliations:** ^1^ Laboratory of Functional Physiology and Valorization of Bio‐Resources of the Higher Institute of Biotechnology of Beja University of Jendouba Jendouba Beja Tunisia; ^2^ Laboratory of Olive Biotechnology Centre of Biotechnology of Borj Cedria Hammam‐Lif Tunisia; ^3^ Department of Anatomopathology Abderrahmane Mami Hospital Ariana Tunisia

**Keywords:** avocado oil, bleomycin, inflammation, oxidative stress, pulmonary fibrosis

## Abstract

Pulmonary fibrosis (PF), is a chronic interstitial lung disease, characterized by changes in the alveoli, excessive accumulation of extracellular matrix, persistent inflammation, and oxidative stress. In this study, we aimed to explore the therapeutic effects of avocado oil (Ao) on bleomycin (BLM)‐induced PF. Four this, 24 male rats were divided into four groups (*n* = 6): the first group served as a control, the second served as a fibrotic group, instilled intratracheally only with BLM (2 mg/kg bw), and the remaining groups were treated by gastric gavage with Ao at different doses (3.5 and 5 mL/kg bw) for 25 days after BLM instillation. The fibrosis induction revealed significant alterations, including increased lipid peroxidation and decreased antioxidant enzyme activities such as superoxyde dismutase (SOD) and catalase (CAT), level of thiols group coupled with a high fibrosis score (FS) and an inflammatory index (II), along with excessive collagen deposition in the pulmonary interstitium. Ao treatment reversed all disturbances induced by BLM in oxidative stress parameters and relatively repairs the histological damage caused by BLM by reducing the FS and the II. The antioxidant, anti‐inflammatory and anti‐fibrosis power of Ao, may suggest this last as a promising candidate for the treatment of PF.

## 1 INTRODUCTION

Idiopathic pulmonary fibrosis (IPF) is the most common pathology among chronic diffuse interstitial lung diseases in adults (Bahri et al., 2022). It is a progressive and irreversible diseases of unknown etiology with a high mortality (Bahri et al., 2022). Pathologically, it is characterized by lesions of alveolar epithelial cells, infiltration of inflammatory cells, proliferation of fibroblasts, and excessive deposition of extracellular matrix (Abidi et al., 2022; Wang et al., 2022). The advanced stage of the disease is marked by a deep disruption of lung architecture leading to impairment of mechanical function, gas exchange, and alveolocapillary diffusion (Abidi, Aissani, et al., 2017). This condition remains poorly understood, and numerous hypothetical factors are involved. The interaction of multiple signaling pathways and cytokines, oxidative stress, and excessive inflammation play a significant role in its onset and development. Experimental induction of this disease often involves various agents, with bleomycin (BLM) being the most commonly used (Brinsi et al., 2022). BLM, a glycopeptides‐type antitumor antibiotic derived from *Streptomyces verticillus*, is currently employed as a chemotherapeutic agent for treating specific tumors like squamous cell carcinoma, testicular carcinoma, lymphomas, and malignant pleural effusions (Karshieva et al., 2023; Pacola et al., 2023). Up to now, the only two available therapeutic antifibrotic drugs are nintedanib and pirfenidone, which only slow down the progression of the disease (Ma et al., 2022). Since antiquity, natural products, particularly those of plant origin, have been considered as an inexhaustible source of remedies for a variety of diseases, as evidenced by Egyptian, Chinese and Greek medicines. Among these substances, *Persea Americana*, commonly known as avocado, which is a tropical or subtropical fruit native to Central America and Mexico, the world leader in avocado production. This fruit is a source of fixed oil, which is a valuable source of antioxidants, unsaturated fatty acids, fat soluble vitamins (A, C and D), phytosterols, carotenoids, and polyphenols (Santana et al., 2019). Due to its richness in various bioactive compounds, avocado oil (Ao) was characterized by its anti‐inflammatory and antioxidant properties, commonly used to reduce the risk of cardiovascular diseases, cancer and diabetes (de Oliveira Marques et al., 2022). Hence, the exploration of new therapeutic agents for the prevention and treatment of PF remains a challenge. In this regard the current study focused on examining the possible protective effect of Ao against BLM‐induced PF in rats.

## 2 MATERIALS AND METHODS

### 2.1 Extraction of avocado oil

The avocado pulp is removed, then dried in an oven at 45°C for 24 h to obtain a powder. Subsequently, 10 g of this dried avocado powder is placed in a cellulose cartridge, which is then introduced into a soxhlet with a flask containing 200 mL of hexane, and this extraction process is carried out for 8 h at 70°C. After the extraction, the solvent is removed by vacuum evaporation using a rotary evaporator resulting in the obtainment of Ao (Tan et al., 2018).

### 2.2 Gas chromatographic analysis

Chromatographic separation and identification of Ao components were performed on a gas chromatography apparatus (6890 N, Agilent Technologies, Santa Clara, CA, USA) equipped with a flame ionization detector and capillary column HP‐Innowax (30 m × 0.32 mm × 0.25 m). The amount of each sample injected was 1 μL. Nitrogen, at a constant flow 1.0 mL/min, was used as the carrier gas, and a spilt/spiltless injector was used with a split ratio of 50:1. The injector temperature was 230°C, and the detector temperature was 280°C. The column temperature was programmed according to the following: initial temperature was 150°C for 1 min and then increased 15°C/min to 210°C and maintained for 5 min before being readjusted again 5°C/min to 250°C and then maintained until the end of the analysis, which takes 25 min. Fatty acid methyl esters were identified by comparison with the standard fatty acid methyl esters (Sigma, Livonia, MI, USA) and were quantified as percentages of the total methyl ester peak areas (Abidi et al., 2020).

### 2.3 Animals and treatment

The study involved 24 healthy adult males Wistar rats (*Rattus norvegicus*) (150 ± 20 g body weight) obtained from a pet store affiliated with the Higher Institute of Biotechnology of Beja. The exploitation of the rats was in compliance with the Local Ethics Committee of Tunis University, adhering to guidelines set forth by International Council of Laboratory Animals Science. The animals were raised in standardized conditions, housed in polypropylene cages, provided with food and water ad libitum, and kept at a temperature of 20°C ± 2°C with a 12–12 h light–dark cycle. All experimental procedures were conducted following the recommendations of the Ethics Committee of Tunis University for the ethical care and use of animals, in accordance with the NH guidelines.

The rats were weighed and then randomly assigned to four groups of six animals each one. The first group, Control: G_1_ (*n* = 6), served as the normal group and did not receive any treatment. The second group: G_2_ (*n* = 6), served as the positive control and underwent fibrosis induction using BLM only (2 mg/kg bw). The third group: G_3_ (BLM+ Ao1, *n* = 6), underwent fibrosis induction and treatment with Ao at a dose of 3.5 mL/kg bw by gastric gavage during 25 days. Similarly, the fourth group: G_4_ (BLM+ Ao2, *n* = 6), received BLM installation followed by a 25 days treatment with Ao at a dose of 5 mL/kg bw (Flores et al., 2019).

For fibrosis induction, the rats were anesthetized by intraperitoneal injection of a solution of sodium pentobarbital (75 mg/kg bw) (Sandoz laboratory, Levallois‐Perret, France). Each anesthetized rat was immediately suspended from a gallows. Fibrosis induction was performed by intratracheal instillation of 2 mg/kg bw of BLM (Bleomycin®, Laboratories Aventis, France) in 200 μL saline, through a sterile catheter (Abidi et al., 2019).

During the experiment (25 days), the body weight of rats was measured every 5 days and the weight gain of rats in each group was compared.

### 2.4 Organ sampling and evaluation of oxidative stress

At the end of the experiment (after 25 days of Ao treatment), the animals were euthanized by the injection of a lethal dose of sodium pentobarbital (200 mg/kg bw Nembutal ceva Animal Health), and various samples were collected (liver and kidneys). Sections of the diaphragm and anterior chest allowed us to extract the heart‐lung block. The same samples were homogenized in a Tris‐NaCl buffer (pH = 7.6) using Ultra‐Turrax. The obtained homogenates were then centrifuged at 1500 rpm at 4°C for 15 min, and the supernatant was collected and transformed into Eppendorf tubes, which were stored at −20°C for subsequent biochemical analyses.

The lung was fixed by intratracheal injection of a 10% formalin solution and immersed in it for 48 h before histological examination. Furthermore, the liver and kidneys were excised and utilized for the same examination.

### 2.5 Body weight variation

During the experiment, the body weight of rats was measured every 5 days, and the mean of weight gain of rats in each group was compared.

### 2.6 Determination of lipid peroxidation

The lipid peroxidation of various organs was assessed by measuring the MDA levels using the method described by Draper and Hadley (1990), based on the coupling of MDA and thiobarbituric acid et 80°C (Draper & Hadley, 1990). in brief, centrifuged hepatic, renal and pulmonary homogenates were mixed with a solution of BHT‐TCA containing 1% BHT dissolved in 20% TCA and centrifuged at 1000 rpm for 10 min at 4°C. Subsequently, the supernatant was collected, to which a solution of 0.5 N HCL and Tris‐TBA solution were added and then heated at 80°C for 10 min. After the samples cooled, the optical density was measured at a wavelength of 535 nm using a spectrophotometer. The absorbance is proportional to the amount of MDA formed, providing an accurate assessment of membrane lipid peroxidation.

### 2.7 Determination of antioxidant enzymes activity

The total activity of superoxide dismutase was mesured following the method of Mirsa and Fridovich (1972), based on the inhibition of epinephrine auto‐oxidation in the presence of SOD (Misra & Fridovich, 1972). At a basic pH, the superoxide anion induces the auto‐oxidation of epinephrine, forming asenochrome. In this process, superoxide dismutase (SOD) competes by inducing the formation of hudrogen peroxide (H_2_O_2_) from the superoxide anion. Catalase is used to catalyze the conversion of H_2_O_2_ to water to prevent the reformation of the superoxide anion. One unit of SOD is difined as the amount of enzymz extract that reduces the rate of adenochrome formation by half. For the experiment, an enzyme extract was added to 2 mL of a reaction mixture containing 10 μL of bovine catalase (CAT, 0.4 U/mL), 20 μL of epinephrine (5 mg/mL), and a carbonate/bicarbonate buffer at 62.5 mM (pH = 10.2). changes in absorbance were mesured at 480 nm.

The catalase activity was determined using the Aebi method in 1974, which is based on the utilization of H_2_O_2_ as a substrate, undergoing degradation into H_2_O and O_2_ (Aebi, 1984). The reaction mixture consisted of 33 mM H_2_O_2_ in 50 Mm phosphate buffer with a pH of 7.0 and the catalase activity was determined by applying an extinction coefficient of 40 Mm/cm for H_2_O_2_.

### 2.8 Determination of thiol groups (‐SH)

The method of Ellman (1959) was used to dertmine th amount of thiol groups (Ellman & Lysko, 1979). Briefly, 50 μL of biological samples (lung, liver, and kidney) was suspended in 1 mL of buffer containing tris base (0.25 M) and EDTA (20 mM) in a basic medium (pH = 8.2). the mixture was vortexed and absorbance dertmined at 412 nm, the A1 value. Next, 20 μL of DTNB (10 mM) dissolved in methanol was added. After incubation, for 15 min at room temperature, a new absorbance measurement was carried out, giving the A2 value. The blank tube contained DTNB only (1050 μL of buffer and 20 μL DNTB). The absorbance value of the blank is B. the qantity of thiol groups is dertmined by the following formula: Thiol group (mM) = (A2−A1)−B × 1.57.

### 2.9 Protein determination

Proteins were quantified using the Bradford method, based on the chemical reaction between the amine group (NH_2_) of protensand the Coomassie blue present in the Breadford reagent, resulting in a blue coloration (Bradford, 1976). In summury, 10 μL of the extract was mixed with 200 μL of Bradford solution. Subsequently, 790 μL oh H_2_O was added, followed by an incubation for 15 min at room temperature. The optical density was measured at a wavelengh of 595 nm. A calibration curve was established using a BSA stock solution. The protein concentration of the samples was determined by refercing the optical density values to the colibration curve established using BSA as a reference.

### 2.10 Histopathological examination

For the histopathological examinaton, rat's left lungs were fixed with formalin dehydeated in a graded series of ethanol, embedded in paraffin, cut into 1 cm^3^ thick serial sections, and stained with hematoxylin and eosin (H&E) to detect inflammatory cells and Masson's trichrome to identify collagen deposition. The evaluation of inflammation and fibrosis score was carried out using a blinded semi‐quantitative system to assess the extent and severity of fibrosis in the lung parenchyma. The severity of fibrosis was assessed with the semi‐ quantitative Ashcroft score, as modified by Ralf‐Huber et al. (Ashcroft et al., 1988; Hubner et al., 2008) Grade 0: normal lung, Grade 1: minimal fibrous thinckening of alveolar or bronchiol walls, Grades 2 and 3: moderate thichning of walls wirthout obvious damage to lungs architecture, Grades 4 and 5: increased fibrosis with definite damage to lung architecture and formaton of fibrous bands or small fibrous mass, Grades 6 and 7: severe distortion of structure and large fibrous areas, honeycomb lung, and Grade 8: total fibrotic obliteration of the field. The determination of the inflammatory index is based on the notation of inflammatory infiltration (set of cellular elements present in an inflammatory focus: histiocytes, fibrocytes, fibroblasts, mast cells) according to a subjective semi‐quantitative scale based on the intensity and location of the inflammation. All lung tissues extracted from the different studied rats were prepared and used in this histological study. Subsequently, each histological section obtained is divided into four reading fields and thus by referring to a 10× microscopic magnification, the final score was expressed as a mean of individual scores observed on all microscopic fields. The inflammatory index is usually determined based on the spatial distribution, the inflammation would be thus, peribronchial, interstitial, or alveolar and the density will be qualified:

Grade 0 = absence of inflammation, Grade 1 = minimal inflammation, Grade 2 = minimal to moderate inflammation (punctate inflammatory infiltrate), Grade 3 = moderate inflammation with thickening of alveolar walls, Grade 4 = moderate to severe inflammation and Grade 5: severe inflammation, massive infiltrates occupying large areas with the presence of follicles (Abidi, Robbe, et al., 2017).

### 2.11 Statistical analysis

Experimental data was performed with IBM SPSS 25.0 software for statistical analysis. Data were presented as mean ± standard error of the mean (SEM). Statistical differences between the groups were analyzed with one‐way analysis of variance (one‐way ANOVA) followed by Bonferroni's post hoc multiple comparison test. *p* < 0.05 was considered statistically significant.

## 3 RESULTS

### 3.1 Fatty acid composition in avocado oil

**FIGURE 1:**
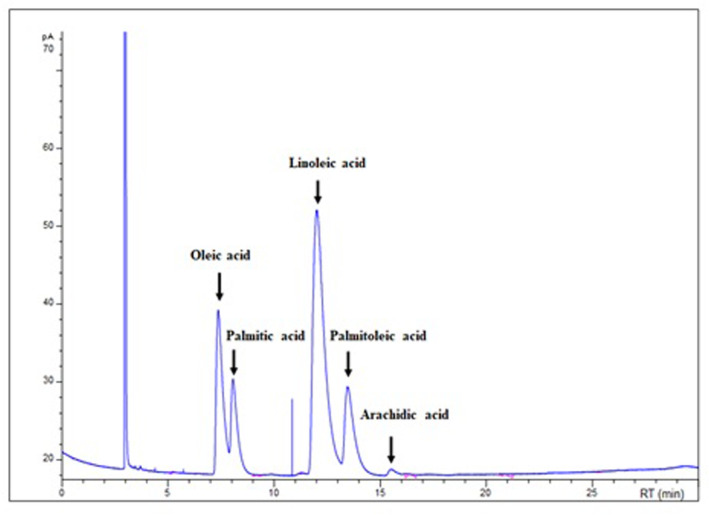
Gas chromatography analysis of fatty acids of avocado oil.

The fatty acid content and composition of Ao was assessed using Gas Chromatography (GC) (Figure 1). Fourteen fatty acids were identified, of which the main detected compounds, their molecular weight, retention time and surface percentage are presented in the Table 1. This biochemical characterization showed the richness of this oil in Oleic, Palmitic, Linoleic, and Palmitoleic acids (56.67%, 18.94%, 13.86%, and 8.78%, respectively).

**TABLE 1:** Fatty acid profile (% total fatty acids) of the total lipids of Avocado oil.

Compounds	Molecular weight	Retention time	Area %
Myristic acid (C14:0)	228.37 g/mol	5.203	0.06
Palmitic acid (C16:0)	256.42 g/mol	7.355	**18.94**
Palmitoleic acid (C16:1)	254.41 g/mol	8.055	**8.78**
Margaric acid (C17:0)	270.46 g/mol	8.985	0.06
Margaroleic acid (C17:1)	268.45 g/mol	9.249	0.04
Stearic acid (C18:0)	284.48 g/mol	11.290	0.02
Oleic acid (C18:1)	282.47 g/mol	12.013	**56.67**
Linoleic acid (C18:2)	280.45 g/mol	13.457	**13.86**
Arachidic acid (C20:0)	312.54 g/mol	15.526	0.94
Alpha‐linolenic acid (C18:3)	278.44 g/mol	16.189	0.15
Eicosenoic acid (C20:1)	310.53 g/mol	16.690	0.12
Behenic acid (C22:0)	340.61 g/mol	20.796	0.07
Erucic acid (C22:1)	338.60 g/mol	21.250	0.24
Lignoceric acid (C24:0)	368.68 g/mol	25.294	0.04

### 3.2 Mortality and morbidity

No rat deaths were recorded during the exprimental protocol, either before or after fibrosis induction. The morbidity of the rats showed a gradual regression until the end of the treatment with Ao. Additionally, the rats regained typical eating and drinking behaviors indicative of their normal state. Importantly, the administration of Ao through gavage did not reveal any signs of toxicity or adverse effects.

### 3.3 Effect of Ao treatment on body weight variation

**FIGURE 2:**
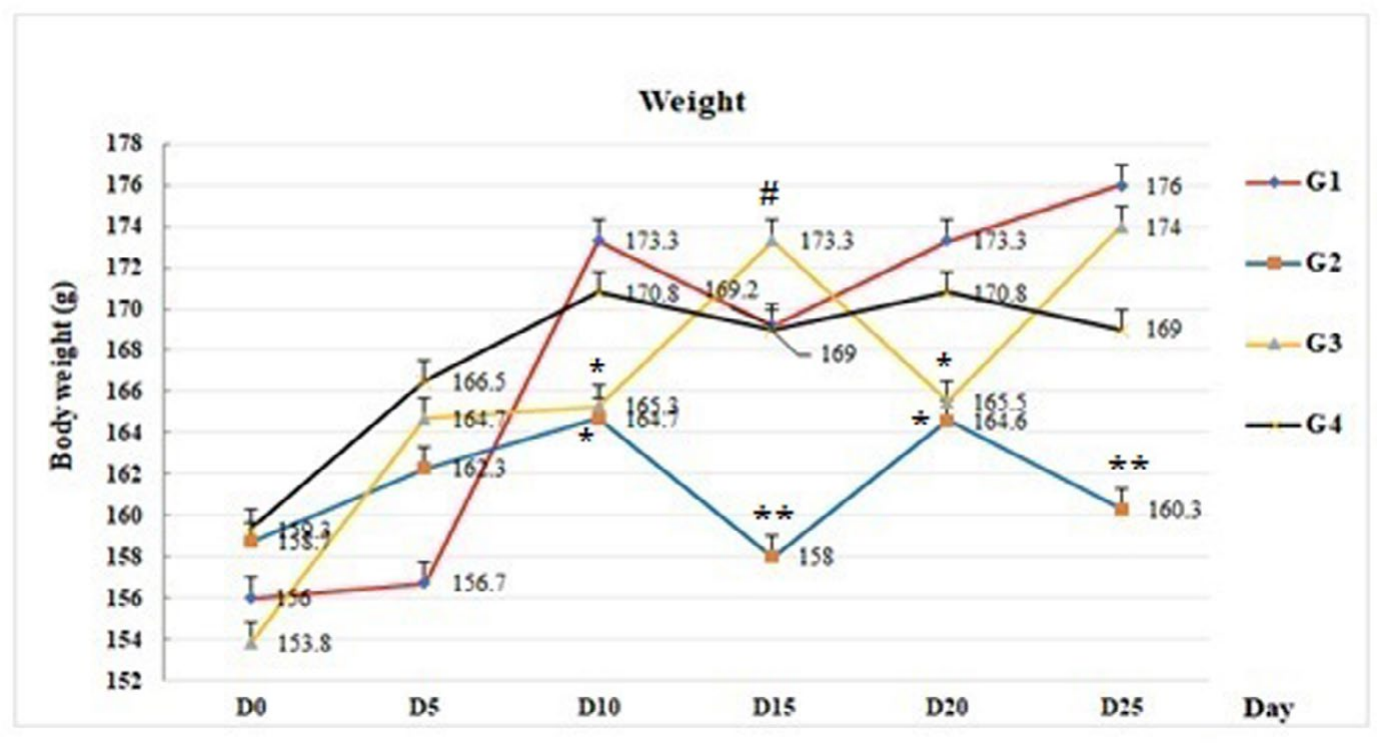
Influence of the administration of Avocado oil on body weight monitoring. G_1_, control group; G_2_, bleomycin group; G_3_, BLM + Ao1 and G_4_, BLM + Ao2 (*n* = 6, X ± SD). *p < 0.05 and **p< 0.01 versus control group (G_1_). #*p*< 0.05 versus G_1_, G_2_ and G_4_.

During the treatment period, the body weights of all rats in the experimental groups were measured each 5 days. The obtained results revealed that administration of BLM at a dose of 2 mg/kg bw yielded a decrease in weight in G_2_ and G_3_ groups compared with the control group observed from day 10 onwards. G_3_ showed another average weight drop around day 20 of treatment. However, in the G_4_ group over a period of 25 days, the body weight loss was attenuated (Figure 2).

### 3.4 Effect of Ao on lipid peroxydation

**FIGURE 3:**
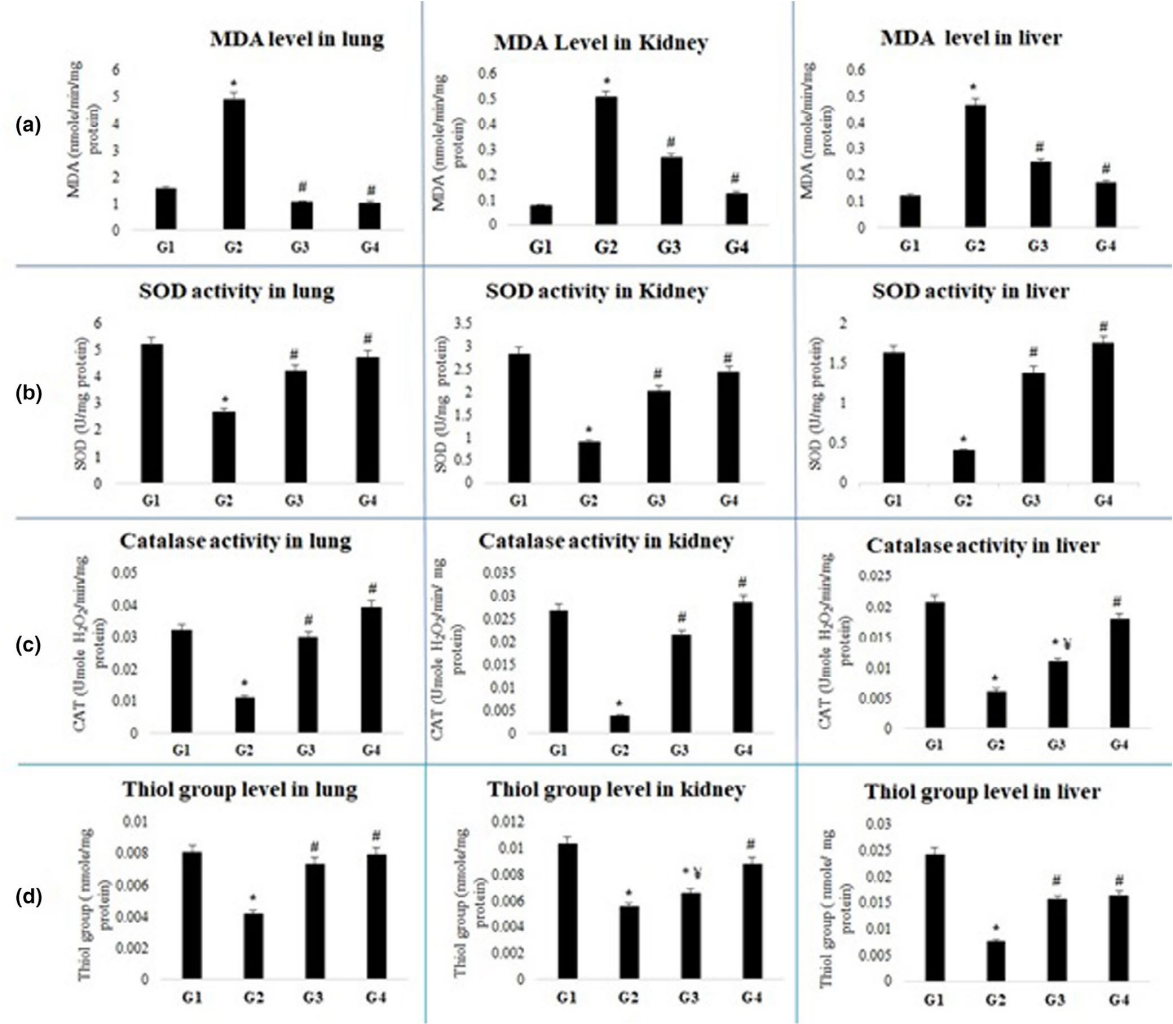
Effects of avocado oil and/or bleomycin treatment on lungs, kidneys, and liver malondialdehyde levels (a); on lungs, kidneys, and liver superoxide dismutase activities (b); on lungs, kidneys, and liver catalase activities (c); on lungs, kidneys, and liver sulfhydryl groups levels (d). G_1_, negative control group; G_2_, bleomycin group; G_3_, BLM + Ao1; G_4_, BLM + Ao2. Values are expressed as means ± SD; number of rats: *N* = 6. *p < 0.05 versus control (G_1_), #*p* < 0.05 versus BLM group (G_2_), ¥*p* < 0.05 versus BLM + Ao2 groups (G_4_); organs were collected after 25 days of treatment.

The administration of BLM significantly increased the lipid peroxidation proven by increased levels of MDA in the pulmonary interstititium. The results of this study, presented in Figure 3a, show that the level of MDA in the lungs is considerably higher in the BLM group (5.36 ± 0.96 nmol/mg protein) compared with the conrol group (1.10 ± 0.96 nmol/mg protein). However, treatment with Ao at the two choised doses (3.5 and 5 mL/kg bw) significantly restored this parameter and brought it closer to the values of control group (2.16 ± 0.29, 2.02 ± 0.24 nmol/mg protein, respectively vs. 1.10 ± 0.43 nmol/mg protein). Smilarly, in kidney and liver tissues, a signifcant increase in MDA levels was observed in the BLM group compared with the control group. In contrast, treatment with Aod showed a significant correction of lipid peroxidation (Figure 3a).

### 3.5 Antioxidant enzymes activities measurement

The pulmonary damage of rats caused by BLM was confirmed by a significant reduction in antioxidant enzymes, in particular Catalase (CAT) and Superoxide Dismutase (SOD). Nevertheless, treatment with Ao signficantly reversed this disturbance. Our results indicated a significant decrease in CAT enzymatic activity in kidney, liver and lung tissues following the onest of BLM when compared with the control group (in lung tissue: 0.010 ± 0.002 μmoL H_2_O_2_/min/mg protein vs. 0.039 ± 0.005 μmoL/min/H_2_O_2_/mg protein, respectively). Treatment with Ao at a dose of 3.5 mL/kg bw resulted in a restoration of this level close to the contol group (0.03 ± 0.002 μmol H_2_O_2_/min/mg protein vs. 0.039 ± 0.005 μmol/min/H_2_O_2_/mg protein, respectively) (Figure 3c). These results were also observed with the second dose of Ao (5 mL/bw). In this same contexte, BLM, also reduced SOD enzymatic activity in lung tissue compared with the control group (1.72 ± 0.02 vs. 5.94 ± 0.03 μ/mg protein). Treatment with Ao at a doses of 3.5 mL/kg bw and 5 mL/g kbw restored SOD activity in lung tissue compared with the control one (4.24 ± 1.01, 4.73 ± 0.350 vs. 5.95 ± 0.03 μ/mg protein). The variations observed are consistent with those recorded in kidney and liver tissue (Figure 3b).

### 3.6 Measurment of thiol group levels

Tissues simples from lungs, kidneys and liver showed a significantly lower levels of thiol groups in the BLM group compared with control group (0.004 ± 0.003 vs. 0.008 ± 0.003; 0.005 ± 0.002 vs. 0.01 ± 0.004; 0.008 ± 0.005 vs. 0.024 ± 0.005, in lung, kidney and liver tissues respectively). In contrast, the Ao treated groups restored thiol groups to those of control group in all the examined tissues (Figure 3d).

### 3.7 Protein determination

The measurement of protein content in various tissues, according to the Bradford method, clearly highlighted a significant reduction in preotein levels in the BLM group samples due to the administration of BLM. Conversely, treatment with Ao at different doses resulted in an increase in these levels, even higher than those of the control group (G_1_) (Table 2).

**TABLE 2:** Effect of Avocado oil (Ao) and/or bleomycin treatment on fibrosis score, inflammatory index, and tissue protein content.

Groups	G_1_	G_2_	G_3_	G_4_
Fibrosis Score	0.0 ± 0.0	6.87 ± 0.13*	4.0 ± 0.09^#^	3.0 ± 0.11^#^
Inflammatory Index	0.0 ± 0.0	5.53 ± 0.46*	3.0 ± 0.31^#^	2.5 ± 0.24^#^
Protein content (g/100 g tissue)				
Lung	29.39 ± 0.10	23.30 ± 0.25*	30.1 ± 0.33	30.59 ± 0.85
Kidney	31.54 ± 1.64	29.54 ± 0.49	32.20 ± 0.89	34.43 ± 0.77
Liver	39.62 ± 1.64	37.07 ± 0.58	41.33 ± 1.83	46.02 ± 1.17^#^

*Note*: Values are expressed as means ± SD; number of rats: *n* = 6.

Abbreviations: G_1_, negative control group; G_2_, bleomycin group; G_3_, BLM + Ao1; G_4_, BLM + Ao2.

* *p* <0.05 versus control.

^#^ *p* < 0.05 versus BLM and control groups.

### 3.8 Histological analysis

**FIGURE 4:**
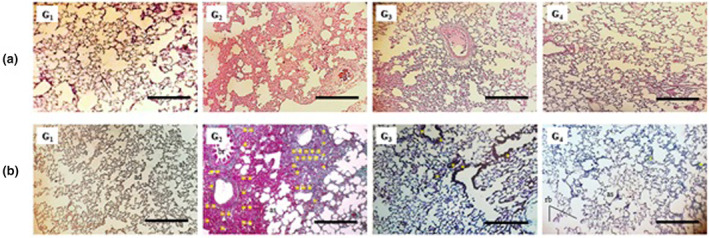
Evolution of histological alterations in rat lungs after BLM instillation and/or Avocado oil treatment (a) Hematoxylin and eosin–stained lung tissue (magnification, ×100; scale bar, 10 μm). (b) Masson's trichrome‐stained lung tissue; collagen is stained in blue and cells in red (magnification, ×100; scale bar, 10 μm). G_1_, negative control group; G_2_, bleomycin group; G_3_, BLM + Ao1; G_4_, BLM + Ao2 (*n* = 6, X ± S); ad, alveolar duct; al, alveoli; as, alveolar sac; br, bronchus; rb, respiratory bronchioles; tb, terminal bronchioles; yellow star (*) = inflammatory infiltrate. Lung tissues were collected after 25 days of treatment.

The evolution of histological alterations in the lungs of rats following the development of fibrosis was determined through two stainings: H&E staining Masson's trichrome. Microscopic observation of the lungs in the control group reveals a normal pulmonary architecture with normal alveolar spaces and a typical thickness of alveolar septa. In contrast, the lung tissues of the fibrotic group (BLM) are characterized by inflammatory infiltration, distruption of cellular architecture, significant thickening of alveolar walls, and the presence of honeycomb structures (Figure 4).

Treatment with Ao partially reduced the severity of BLM‐induced lung lesions, with varying degrees depending on the administred dose. The Ao treated group (5 mL/kg bw) showed improvement in pulmonary tissue, characterized by focal inter‐alveolar fibrosis with low intra‐alveolar inflammatory infiltration.

For Masson's Trichrome staining, the sections confirm the H&E results, with the maintaining tissue structure, displaying a thin layer of collagen on the bronchial wall in the control group. In contrast, the BLM group confirms the establishment of fibrosis in lung tissue, justified by a significant collagen depostion un the bronchiol wall and within the inter and intra‐alveolar septa. Results from the Ao‐treated groups indicate an improvement in collagen distribution, localized with preservation of tissues in some pulmonary regions, especially within the inflammatory infiltrate.

The severity of fibrosis was assessed using a semi‐quantitative scoring system. A significant increase in the fibrosis score was observed in the BLM group compared to the control one. However, in the Ao‐treated groups, this score decreased significantly compared with the BLM group (Figure 4 and Table 2).

Intratracheal administration of BLM caused severe inflammation, resulting in a significant increase in the inflammatory index in the BLM group compared with the control group. On the other hand, treatment with Ao at different doses resulted in a significant reduction in the inflammation index compared with BLM group (Figure 4 and Table 2).

## 4 DISCUSSION

PF is a progressive and incurable lung disease characterized by the excessive formation of scar tissue in the lung leading to a decline in lung function. The inflammation and oxidative stress play a crucial role in the development of FP. Moreover, the inflammatory response, indicated by high levels of pro inflammatory cytokines and inflammatory cells, contributes to lung tissue damage and fibrosis. Oxidative stress, caused by an imbalance between the production of reactive oxygen species (ROS) and antioxidant defenses, leads to cellular damage and activates fibrotic pathways (Abidi et al., 2022; Elhady et al., 2022; Ommati et al., 2023; Roksandic Milenkovic et al., 2022; Zhang et al., 2023). In the present study, we aimed to investigate the therapeutic effect of Ao on BLM‐ induced PF in rats. The choice of this oil based on its high vitamin D content. The last is known for its potential effects on the immune system, reducing inflammation and modulating tissue healing processes (Wu et al., 2022). In relation to PF, where inflammation and excessive scarring are key features, vitamin D may offer potential therapeutic improvements (Magro‐Lopez et al., 2022). This vitamin is also involved in regulating antioxidant response and may contribute to reducing oxidative stress, a factor in the progression of PF (Sepidarkish et al., 2019).

Pervious experimental studies on PF have associated the onset of this significant variations in body weight. Indeed, a remarkable decrease in body weight was observed after intratracheally BLM instillation, reflecting the damage resulting from inflammation and the general state of stress caused in the BLM group (Abidi, Robbe, et al., 2017). On the other hand, PF has frequently been linked to a dysregulation of the antioxidant/oxidant balance, induced by the ROS throughout the body, particularly in lung, kidney, and liver (Abidi et al., 2022). In this respect, according to previous literature studies, spontaneous resolution after Day 21 of intratracheal injection of BLM has been confirmed: from this period, fibrosis begins to resolve in most cases, especially in mice (Ruscitti et al., 2017). This is related to active tissue remodeling, inhibition of myofibroblast activity and progressive degradation of excess extracellular matrix. This spontaneous resolution remains a limitation for the evaluation of therapies targeting chronic stages, and this is what led us to limit the duration of Ao treatment to 25 days. Furthermore, in this study, weight evolution evolve in the same way in all groups except for the specific periods (Day 10 for G_2_ and days 10 and 20 for G_3_) and thus, no evidence of spontaneous resolution could be confirmed.

On the other hand, our results revealed an oxidative stress state thrust by the BLM in lung tissue, reflected by a decrease in the antioxidant enzymes activities such as the SOD (1.72 ± 0.02 vs. 5.94 ± 0.03 μ/mg protein), which represents the main intracellular antioxidant line of defense against free radicals by catalyzing the dismutation of superoxide ion into H_2_O_2_ through the release of molecular oxygen (Stephenie et al., 2020). Furthermore, CAT activity was charactirized by a significant reduction in the BLM group compared to the control one (0.010 ± 0.002 vs. 0.039 ± 0.005 μmole H_2_O_2_/min/mg protein), this enzyme contiubutes to the conversion of H_2_O_2_ into water and oxygen. In the same contexte, a decrease in the level of thiol group was observed (0.004 ± 0.001 vs. 1.10 ± 0.43 nmole/min protein), as well as an increase in the level of MDA (5.36 ± 0.96 vs. 1.10 ± 0.43 nmole/min/mg protein). This oxidative disturbances were also observed in hepatic and renal tissues. Also, these oxidative perturbations are consistent with the results of the literature, in which the cytotoxic of BLM causes an excess formation of ROS, which involve the oxidation of iron II to iron III, inducing a reduction of oxygen to free radicals (Abidi et al., 2019; Abidi, Robbe, et al., 2017; Bahri et al., 2020). The intracellular formation of ROS leads to the attack of cells membranes by the degradation of polyinsatured fatty acids, resulting in the formation of MDA, which explains the increase in MDA following exposure to BLM. The generation of ROS can also cause damage to cells organelles, so that intracellular antioxidant enzymes lose their ability to fight oxidative stress (Abidi et al., 2020). The overall imbalance between the oxidant/free radical in the various tissues studied was confirmed by histological observations which showed that the BLM group exhibited remarkable alterations in lung architecture, characterized by severe structural lesions in the alveoli, significant infiltration of immune cells, notable hyperplasia of fibroblasts, significant enlargement of the alveolar septum and excessive accumulation of collagen fibers. This state is the results of the activation of fibroblasts and their differanciation into myofibroblasts, which is a crucial stage in the developemnet of FP, causing thickninng of the ECM and inhibation of its degradation, that leads to hardening of the alveolocappillary wall, resulting in breathing difficulties and reduced pulmonary compliance (Ortiz‐Zapater et al., 2022).

Ao treatment significantly reduced MDA levels, indicating a reduction in oxidative stress and subsquent lipid peroxidation. It is thought that this effect of Ao is linked to its free radical scavenging effect and antioxidant activity, which reduces oxidative stress caused by BLM via the generation of ROS. At a dose of 5 mL/bw, Ao showed a pronounced efficacy against oxidative stress, as evidanced by the increase in antioxidant enzymes activity, thiol group and MDA levels. These benefical effects can be attributed to the bioactive substances present in this oil, such as vitamin D, carotenoid, phytosterols, polyphenols and above all, fatty acids, especially Oleic, Palmitic, Linoleic, and palmitoleic acids. Thes later are associated with various therapeutic effects, including antioxidant and anti inflammatoty activity, cancer prevention and modulation of angiogenesis (Garcia‐Berumen et al., 2022; King‐Loeza et al., 2023). Oleic acid (mono insatured acid) is known for its benefits against cardiovascular diseases, it acts as a powerful immunomodulator and anti‐inflammatory, especially by stimulating phagocytosis through the activation of enzymes which produce superoxide molecules (O_2_^−^), this partly explains increases in SOD levels in tissues of Ao‐treated groups. Palmitic acid (saturated), Linoleic and palimitleic aicids are very effective as a hypotensive vasodilator, generally involved against inflammation and stress states and maintaining balanced cholesterol content (Djuricic & Calder, 2021). This richness in these fatty elements probably gives this oil its ameliorative capacity on the state of oxidative stress caused by BLM, as well as its anti‐inflammatory effects, especially proven histologically at the pulmonary level by the reduction in fibrosis score and the inflammatory index compared to the lung tissues of rats in the BLM group. Furthermore, the abundance of Vitamin D, oleic, palmitic, linoleic, and palmitoleic acids, all known for their anti‐inflammatory and antioxidant properties, gives this oil its regulatory power within the immune system by reducing the production of pro‐inflammatory cytokines. and by increasing the expression of certain antioxidant enzymes. These beneficial effects are dependent on several other physiological factors as well as the choice of doses and the interaction of these molecules with other active compounds at the cellular and systemic level.

## 5 CONCLUSION

Our study highlights the protective effect of avocado oil against BLM‐induced pulmonary inflammation and fibrosis in rats, as confirmed by various tests and histopathological evaluation. The noticeable improvement in the lung morphology in the groups treated with Ao is significant. This oil reduced lipid peroxidation and increased levels of antioxidants defense enzymes (SOD and CAT), as well as thiol group levels. A significant protective effect of Ao was observed at doses of 5 mL/kg bw. This protective potential of Ao can be attributed to its confirmed antioxidant activity, capable of neutralizing the elimination of oxygen and free radicals, as well as its anti‐inflammatory capacity. However, further research is needed to identify the chemical components responsible for the protective effect of Ao. Additionally, more detailed studies are required to fully understand the molecular mechanisms underlying the protective effect of Ao's active compounds against BLM‐induced lung injuries.

## AUTHOR CONTRIBUTIONS

Naoures Ochi and Anouar Abidi, ensured the preparation of the experimental protocol and its realization, was involved in all the analyzes carried out, wrote the manuscript and interpreted the results obtained. Anouar Abidi, is identified as the guarantor of the paper, taking responsibility for the integrity of the work as a whole, from inception to published article. Wael Taamalli, ensured the chemical analysis of the natural extracts by the GC technique. Ayda Ayedi, read the HE and Trichrome histological sections and is involved in the interpretation of its results. Hichem Sebai, participated in revision and correction of manuscripts. All authors have reviewed and approved the submission of this manuscript version.

## FUNDING INFORMATION

This scientific work was funded by the Tunisian Ministry of High Education and Scientific Research.

## CONFLICT OF INTEREST STATEMENT

The authors declare that they have no competing interests.

## ETHICS STATEMENT

Animals were cared for according to the principles of the local Ethics Committee on Animal Welfare (University of Jendouba: UJ2022‐001‐1083) in accordance with the recommendations of the International Council of Laboratory Animal Science.

## CONSENT FOR PUBLICATION

Not applicable.

## Data Availability

Not applicable.
